# Engineered XylE as a tool for mechanistic investigation and ligand discovery of the glucose transporters GLUTs

**DOI:** 10.1038/s41421-019-0082-1

**Published:** 2019-03-05

**Authors:** Xin Jiang, Jianping Wu, Meng Ke, Shuo Zhang, Yafei Yuan, Jason Ye Lin, Nieng Yan

**Affiliations:** 1 State Key Laboratory of Membrane Biology, Beijing, 100084 China; 2 Beijing Advanced Innovation Center for Structural Biology, Beijing, 100084 China; 3 Tsinghua-Peking Joint Center for Life Sciences, School of Life Sciences and School of Medicine, Tsinghua University, Beijing, 100084 China; 4 Department of Genetics and Complex Diseases, Harvard T.H. Chan School of Public Health, Boston, MA 02115 USA; 5 Present Address: Department of Molecular Biology, Princeton University, Princeton, NJ 08544 USA

**Keywords:** X-ray crystallography, Biological techniques

Dear Editor,

Glucose is the primary energy supply for metabolism and a versatile precursor for biomolecule synthesis. Cellular uptake of glucose mainly relies on two types of glucose transporters, the sodium gradient driven glucose symporter SGLT family and the facilitative uniporter GLUT family. Glucose uptake is markedly elevated in tumor cells to compensate for the less-efficient ATP production via glycolysis, a phenomenon known as the Warburg effect. Overexpression of GLUT family members GLUT1 or GLUT3 has been observed in various types of tumors, making them potential targets for novel drug discovery against cancer^1^.

Structures of GLUT1, GLUT3, and GLUT5 were obtained in distinct conformations, which collectively recapitulate the nearly complete alternating access cycle, elucidate the molecular basis for substrate selection and transport, and shed light on the mechanistic understanding of pathogenic mutations^2–5^. The structures are instrumental for mechanistic investigations and ligand discovery. However, in our attempt to establish an in vitro biochemical characterization and screening system for GLUT1/3, we found that recombinantly expressed GLUT proteins were fragile after detergent extraction, impeding mutational analysis. We thereby employed the xylose:proton symporter XylE from *E. coli*, which is one of the closest bacterial homologs of GLUTs, as a surrogate. In addition to the overall structural similarity, the substrate-binding site is highly conserved between the bacterial and human proteins despite that XylE can only bind to, but not transport glucose^3,6^.

Both GLUTs and XylE contain a central substrate binding site that accommodates the ligand in both outward- and inward-facing states^2,3,6^. However, this primary binding site undergoes minor conformational changes owing to the local structural shift accompanying the switch between the outward and inward conformations. In addition, the structure of maltose-bound GLUT3 reveals a potential secondary glucose binding site on the extracellular side that is lacking on the intracellular side^2,3^. These structural observations suggest that the sugar porter (SP) members may have asymmetric affinities for exofacial (access from the extracellular side, or outward-facing state) and endofacial (access from the intracellular side, or inward-facing) binding, a notion that is supported by previous characterizations of human GLUT1^7,8^.

In this study, we attempt to address two questions. First, does XylE share the same asymmetric binding affinities in outward- and inward-facing conformations like GLUT1? Second, can XylE be employed to distinguish endofacial and exofacial ligands for GLUTs?

To test this proposition, we sought to engineer XylE variants that would exhibit constitutively outward- or inward-facing state. For this purpose, we first carried out inter-domain crosslinking experiment. Based on the structure, we identified two pairs of residues for Cys substitution (Supplementary Fig. S1a). The double mutations V35C/E302C (ExoCC) and A152C/S396C (EndoCC) were predicted to form disulfide bonds on the extracellular and intracellular side, respectively, under oxidative condition, and hence locking the protein in inward facing and outward facing, respectively (Fig. 1a, Supplementary Fig. S1a).

**Fig. 1:**
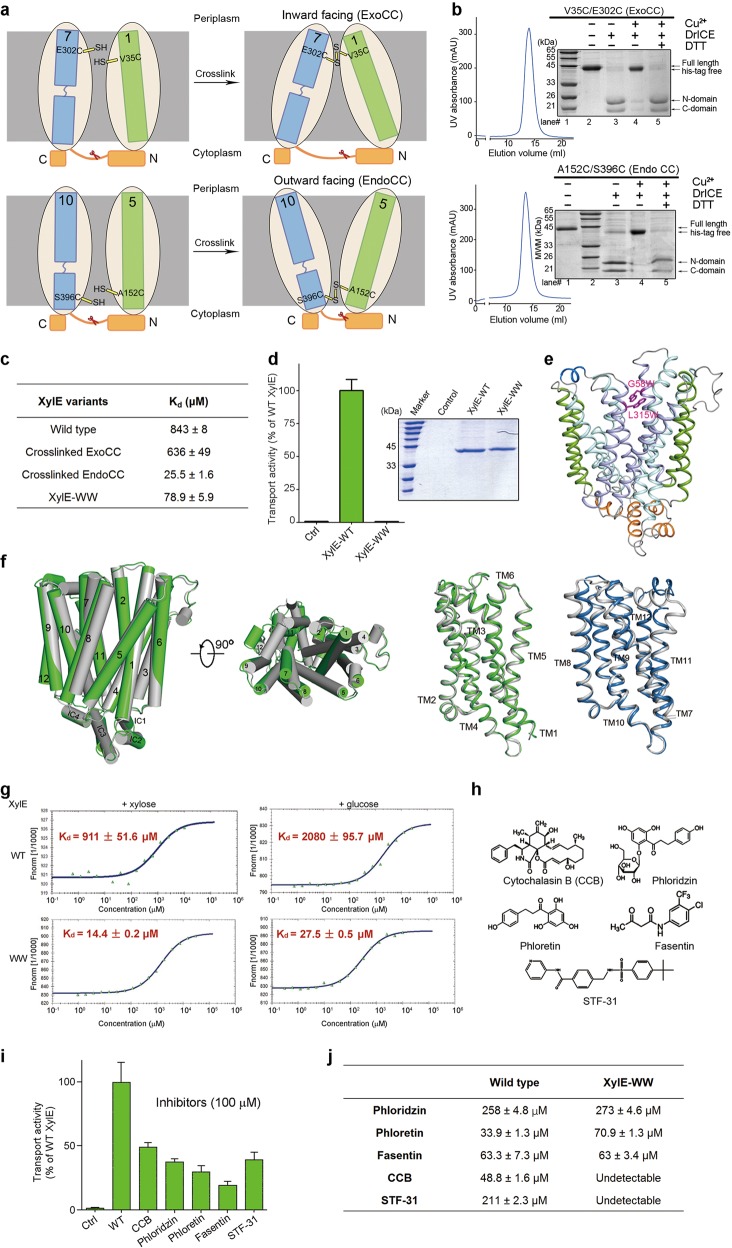
XylE-WW as a surrogate to discriminate exofacial and endofacial ligands for GLUTs. **a** Structure-guided introduction of disulfide bonds to lock XylE in constitutively inward-facing (upper) and outward-facing (lower) conformations. ExoCC: disulfide bond on the extracellular or exofacial side, EndoCC: disulfide bond on the intracellular or endofacial side. In the cartoon diagram, the cysteine mutations for disulfide bond formation are highlighted and a DrICE protease cutting site, indicated by the red scissor in each panel, was introduced for validation of the disulfide-bond formation. In the absence of the indicated disulfide bond, DrICE would cleave both XylE variants to two segments that can be separated on reducing SDS-PAGE. After formation of disulfide bond, the XylE variants would remain to be one band on SDS-PAGE after DrICE cleavage. **b** Biochemical validation of disulfide bond formation. The ExoCC and EndoCC XylE variants were both purified to homogeneity and subjected to DrICE proteolysis under indicated conditions. The distinct patterns on reducing SDS-PAGE with or without reducing agent DTT confirms the formation of designed disulfide bonds. **c** Summary of ITC measurement of the binding affinity between xylose and XylE variants. **d** The XylE-WW lost transport activity. Control group refers to protein-free liposome in the counterflow assay. The transport activity of XylE-WW is normalized relative to that of wild-type (WT) XylE. Error bars represent s.d. **e** The crystal structure of XylE-WW displays an outward-facing conformation. The corresponding TM segments in the four 3-helix repeats in a MFS fold are colored the same, pale purple for TMs 1/4/7/10, pale cyan for TMs 2/5/8/11, and green for TMs 3/6/9/12. The extracellular and intracellular helices are colored marine and orange, respectively. The two introduced Trp residues are shown as magenta sticks. **f** Minor conformational changes between the outward-facing structures of WT XylE and XylE-WW. Left: overall structure comparison between XylE-WW (green) and wild type XylE (gray). XylE-WW structure superposes with wild type XylE structure with r.m.s.d 0.794 Å. Right: structure alignment of N/C domain between XylE-WW and wild-type XylE. Wild-type XylE are colored gray. N domain of XylE-WW is colored green. C domain of XylE-WW is colored marine. The N-domain (residues 5–219) and C-domain (residues 277–462) superpose with wild type XylE with r.m.s.d 0.642 and 0.583 Å, respectively. **g** Outward-facing XylE has higher affinity for d-xylose and d-glucose than WT XylE. The affinities between the XylE variants and the ligands were measured by Microscale thermophoresis analysis (MTS). **h** The chemical structures of the tested inhibitors. **i** The counterflow activity of XylE can be inhibited by representative GLUT1 inhibitors. Each inhibitor was applied at 100 μM, and the remaining transport activity of XylE was normalized against WT XylE in the absence of any inhibitor. **j** Summary of MST measurement of the binding affinity between GLUTs inhibitors and XylE variants in different conformational states. Results were presented by dissociation constant

To verify formation of the designed disulfide bonds, we introduced a DrICE protease-specific cleavage site (DEVDA) into the intracellular helix ICH3 (Fig. 1a, Supplementary Fig. S1b)^9^. In the presence of the disulfide bond, cleavage of the XylE variant by DrICE was predicted to result in two bands in reducing environment, but only one under oxidative condition. Both XylE variants, ExoCC and EndoCC, retained good behavior on size exclusion chromatography before crosslinking (Supplementary Fig. S1c). Purified ExoCC and EndoCC were subject to Cu^2+^ oxidation for disulfide bond formation. The crosslinked XylE variants were examined with gel filtration and SDS-PAGE, which also showed good protein behavior and high crosslinking efficiency. Cleavage by DrICE in both oxidative and reducing conditions confirmed formation of the disulfide bond (Fig. 1b).

The transport activities of the XylE variants were examined in proteoliposome-based counterflow assay before and after crosslinking. Prior to crosslinking, the transport activities of ExoCC and EndoCC XylE were similar to that of wild-type (WT) XylE. As expected, their transport activities were completely lost after crosslinking (Supplementary Fig. S1d).

We then measured the affinities of the constitutively inward- (ExoCC) and outward-facing (EndoCC) XylE variants with the substrate d-xylose using isothermal titration calorimetry (ITC). Whereas the apparent dissociation constant K_d_ for WT XylE is 843 ± 8 μM, those for crosslinked ExoCC and EndoCC are 636 ± 49 and 25.5 ± 1.6 μM, respectively (Fig. 1c, Supplementary Fig. S2). Even though WT XylE and ExoCC share similar free energy changes (*ΔG*), the values for enthalpy change (*ΔH*) and entropic-free energy component (*TΔS*) are markedly different between these two variants. The *ΔH* of wild-type XylE is positive, which makes *TΔS* as sole contribution to *ΔG*. By contrast, crosslinked ExoCC has negative *ΔH*, while *TΔS* contribute to *ΔG* to a lesser degree (Supplementary Table S1). The crosslinked EndoCC also has negative *ΔH* and even a smaller contribution from *TΔS*. These results suggest that crosslinked XylE variants are restricted to certain conformational states and XylE does have asymmetric affinities for substrate binding on the extracellular and intracellular sides.

Because preparing the crosslinked variants was time-consuming, we then sought to engineer XylE mutant that could be locked in certain conformation. A LacY mutant with double Trp substitutions in LacY (G46W, G262W)^10^ exhibited constitutively outward-facing conformation. Based on structural similarity, we introduced two Trp residues on the extracellular segments of TM2 and TM8 (G58W/L315W) and named this double Trp mutant XylE-WW. Counterflow assay showed loss of transport activity of XylE-WW (Fig. 1d). ITC measurement revealed that XylE-WW and EndoCC bound to D-xylose with similar affinities, supporting its outward-facing conformation (Fig. 1c, Supplementary Fig. S2, Table S1).

To further confirm the conformational state of XylE-WW, we determined the crystal structure of XylE-WW at 3.7 Å resolution (Fig. 1e, Supplementary Table S2). The overall structure displays an outward-facing conformational state that is similar to the WT protein (Fig. 1f). The densities for the two introduced Trp residues are clearly discernible in the 2Fo-Fc map (Supplementary Fig. S3a). As TM2 and TM8 are not involved in substrate binding, the two Trp residues prevent closure of the N and C domains on the extracellular domains, but do not affect substrate binding site (Supplementary Fig. S3b).

Next, we examined whether the constitutively outward-facing XylE-WW could act as a surrogate to screen for GLUT inhibitors. When using ITC for affinity measurement, however, DMSO, which is the most commonly used solvent for hydrophobic inhibitors, caused serious noise and impeded data processing. We therefore tried MicroScale Thermophoresis (MST), which monitors the thermophoresis signal of equilibrium state and is insensitive to solvent background, to measure the affinities of XylE variants with known ligands.

The binding affinities between WT XylE and d-xylose were almost identical when measured by ITC and MST. The affinity between XylE-WW and d-xylose was slightly higher by MST measurement than by ITC, 14.4 ± 0.2 vs 78.9 ± 5.9 μM (Fig. 1g, left panels). These results support MST to be used for reliable affinity measurement. Using MST, the apparent affinities for glucose with XylE-WT and XylE-WW are 2080 ± 95.7 and 27.5 ± 0.5 μM, respectively (Fig. 1g, right panels).

Five known GLUT inhibitors, including Cytochalasin B (CCB), Phloretin, Phloridzin, Fasentin, and STF-31, were used to test the feasibility of using XylE variants as surrogate (Fig. 1h). The inhibitory effect of these compounds on XylE was examined in the counterflow assay, and all of them exhibited inhibition of the transport activity of XylE to different extent (Fig. 1i). We then measured their binding to both XylE-WT and XylE-WW. While all five inhibitors can bind to XylE-WT with affinities ranging from 30 to 300 μM, only the exofacial GLUT inhibitors, Phloretin, Phloridzin, Fasentin, bind to XylE-WW^11^ (Fig. 1j, Supplementary Fig. S4). It is noted that unlike d-xylose and d-glucose that bind to both conformations of XylE, the exofacial inhibitors bind to XylE-WT and XylE-WW with similar affinity. On the other hand, interaction between the endofacial GLUT inhibitors CCB and STF-31 with XylE-WW could not be measured by MST^5,12^, consistent with the constitutive outward-facing conformer of XylE-WW.

In sum, our study employing designer XylE variants revealed the asymmetric substrate binding affinities between outward- and inward-facing states of XylE, confirming a conserved mechanism among SP members. Using this asymmetric property in transport cycle, we developed an in vitro assay system that contains different conformational XylE and can be used for preliminary screening of GLUT inhibitors and for discrimination of exofacial and endofacial ligands.

## Accession code

The atomic coordinates and structure factors of XylE-WW have been deposited in the Protein Data Bank under the accession codes 6N3I.

## Supplementary information

Supplementary Information

## Supplementary material

**Supplementary Information** accompanies the paper at (10.1038/s41421-019-0082-1).
