# Supplementary material for: Engineered XylE as a tool for mechanistic investigation and ligand discovery of the glucose transporters GLUTs
Source: Cell Discov. 2019 Mar 5;5:14. doi: 10.1038/s41421-019-0082-1 (PMC6399220; doi:10.1038/s41421-019-0082-1)
Supplement: Supplementary file 1 — Supplementary Information [file 41421_2019_82_MOESM1_ESM.pdf]

Supplementary information

Engineered XylE as a tool for mechanistic investigation and ligand discovery of the glucose transporters GLUTs

Xin Jiang[1,2,3,5], Jianping Wu[1,2,3,5], Meng Ke[1,2,3], Shuo Zhang[1,2], Yafei Yuan[1,2], Jason Ye Lin[4], and Nieng Yan[1,2,3,5, *]

[1]State Key Laboratory of Membrane Biology, [2]Beijing Advanced Innovation Center for Structural Biology, [3]Tsinghua-Peking Joint Center for Life Sciences, School of Life Sciences and School of Medicine, Tsinghua University, Beijing 100084, China

[4]Department of Genetics and Complex Diseases, Harvard T.H. Chan School of Public Health, Boston, MA 02115, USA

[5]Present address: Department of Molecular Biology, Princeton University, Princeton, NJ 08544, USA

[*]To whom correspondence should be addressed. E-mail: nyan@princeton.edu

Materials and Methods

Protein expression and purification

The complementary DNA of full-length XylE from *E. coli* strain O157:H7 was subcloned into modified pET15b vector (Novagen), which contains an additional DrICE-cutting site (DEVDA) between N-terminal his-tag and target protein. All mutants were generated with standard PCR-based strategy. The overexpression and purification of XylE were described elsewhere[1]. Briefly, XylE were overexpressed in *E. coli* BL21 (DE3) cells. Transfected *E. coli* BL21 (DE3) cells were grown in 1 L Luria-Bertani medium within 2 L flasks at 37 ℃. Cell culture was induced by 0.2 mM isopropyl β-D-thiogalactoside (IPTG) when the cell density (validated by OD_{600nm} reading) reached 1.5. After growth for 4 h at 37 ℃, *E. coli* cells were harvested through centrifugation and homogenized with buffer containing 25 mM Tris-HCl pH 8.0 and 150 mM NaCl. Cells were disrupted by sonication and cell debris was removed by low-speed centrifugation. The plasma membrane was harvest by ultracentrifuged at 150,000 g for 1 h. The membrane proteins were extracted from membrane fraction in buffer containing 25 mM Tris-HCl pH 8.0, 150 mM NaCl and 1.5% (w/v) dodecyl-β-D-maltopyranoside (DDM; Anatrace) at 4 ℃ for 1 h. The debris of cell membrane was removed by another ultracentrifugation step at 150,000 g for 30 min. The supernatant was applied to Ni^{2+}-nitrilotriacetate affinity resin (Ni-NTA; Qiagen). After resin was rinsed with buffer containing 25 mM Tris-HCl pH 8.0, 150 mM NaCl, 20 mM imidazole and 0.02% DDM, the protein was eluted from the affinity resin with buffer containing 25 mM Tris-HCl pH 8.0, 150 mM NaCl, 250

mM imidazole and 0.02% DDM. The elution was concentrated and further purified with gel filtration (Superdex-200; GE Healthcare) in various buffer for different usage. The peak fractions from gel filtration were collected and flash-frozen in liquid nitrogen for further experiments.

Crystallization, data collection and structure determination

XylE-WW protein was purified by gel filtration in buffer containing 25 mM Tris-HCl pH 8.0, 150 mM NaCl and 0.056% 6-Cyclohexyl-1-Hexyl-β-D-maltoside (Cymal-6; Anatrace). Crystals of XylE-WW were grown at 18 ℃ by the hanging-drop vapor diffusion method by mixing 1 μl protein with 1 μl reservoir buffer containing 0.1 M NaCl, 0.1 M Li_2SO_4, 0.1 M MES pH 6.5, 30% PEG400 (v/v). Crystals appeared in 2-3 days and grew to full size in about 1 week. The crystals were directly flash-frozen in a cold nitrogen stream at 100 K for further data collection.

The data sets were collected at SSRF beamline BL17U and integrated and scaled using HKL2000[2]. Further processing was carried out using CCP4 suite[3]. The phase was solved by molecular replacement using PHASER[4] with XylE (PDB code: 4GBY) as a searching model. The model was further rebuilt in COOT[5] and refined with PHENIX[6]. Data collection and structure refinement statistics are summarized in Supplementary Table S2.

Cysteine crosslinking

XylE cysteine mutants (A152C/S396C, V35C/E302C) were overexpressed and purified with same methods as described previously. After protein was eluted from Ni^{2+}-NTA affinity chromatography column, 1.5 mM $CuCl_2$ was added into protein elution to initiate crosslinking. The crosslinking system was shaken at room temperature for 2 h. Gel filtration with Superdex-200 column was followed to remove the Cu^{2+}. After gel filtration, the crosslinked XylE protein was transferred to buffer containing 25 mM MES pH 6.5, 150 mM NaCl and 0.056% Cymal-6 for further isothermal titration experiment.

The validation of crosslinking was conducted through DrICE protease digestion. The crosslinked protein was mixed with DrICE protease with ratio 100:1 (w: w). Then the mixture was incubated at room temperature for 3 h. After digested protein was reduced by 200 mM DTT for 10 min at room temperature. These protein samples were applied to SDS-PAGE to exam whether they were crosslinked.

Preparation of liposomes and proteoliposomes.

The proteoliposomes were made by following steps. *E. coli* polar lipids (Avanti) were dissolved in chloroform/methanol mixture (3:1, v/v) and dried with nitrogen gas. Then the lipids were dissolved to 20 mg/ml with KPM 6.5 buffer (50 mM potassium phosphate, 2 mM magnesium sulfate, pH 6.5) and 20 mM D-xylose. The liposomes were homogenized with 5 rounds freeze/thaw cycles and extrusion. Then liposomes were incubated with 1% n-octyl-β-D-glucoside (β-OG; Anatrace) at 4 ℃ for 30 min. After that, liposomes were mixed with 200 μg/ml XylE or mutants protein and

incubated for another 1 h at 4 ℃. After protein was integrated into liposomes, β-OG was removed by incubation overnight with 400 mg/ml Bio-Beads SM2 (Bio-Rad). Then the proteoliposomes were frozen and thawed for 5 times and extruded with 0.4 μm membrane filter (Millipore) for 21 time. The homogenized proteoliposomes were ultracentrifugated at 100,000 g for 1 h and rinsed with ice-cold KPM 6.5 buffer for 2 times to remove extra sugar. Finally, the proteoliposomes were resuspended in ice-cold KPM 6.5 buffer to 100 mg/ml before the counterflow assay. Control liposomes were made with the same protocol except for protein insertion step.

Counterflow assay

The counterflow assays were performed at 25 ℃. For each assay, 2 μl 100 mg/ml proteoliposomes preloaded with 20 mM D-xylose were mixed with 100 μl KPM 6.5 buffer containing 1 μCi of D-^{3}H-xylose (American Radiolabeled Chemicals, Inc.). The specific radioactivity of D-^{3}H-xylose was 20 Ci/mmol and final concentration of the external D-^{3}H-xylose was 0.5 μM. The uptake of radiolabeled substrates was allowed for 30 s. Then proteoliposomes were rapidly filtered with 0.22 μm filters (Millipore) and washed with 2 ml ice-cold KPM 6.5 buffer. The filter was then taken for liquid scintillation counting. All experiments were repeated for three times. Error bars represent s.d.

Isothermal titration calorimetry

The binding affinity between wild type XylE and xylose was measured with an ITC200 micro calorimeter (MicroCal). The wild type XylE and XylE mutant proteins were purified as previous description. In gel filtration step, all the XylE protein for ITC test was transferred to buffer containing 25 mM MES pH 6.5, 150 mM NaCl and 0.056% Cymal-6. The peak fractions were pooled and concentrated by Centricon (50 kDa cutoff; Millipore) to 0.1 mM. D-xylose was dissolved in the same buffer to different concentrations. XylE proteins were titrated by D-xylose at 22 ℃. The titration system was stirred continuously at 500 rpm. The heat of dilution was measured through titrating ligand into sample buffer without protein. The heat flow tracings were integrated and the heat of dilution was deducted. The associate constant K_a and enthalpy change ΔH were determined by the isotherm directly. Then the free energy change ΔG was calculated through equation $\Delta G = -RT \ln K_a$ and the entropy change ΔS was calculated by $\Delta G = \Delta H - T\Delta S$. In these equations, R is the gas constant and T is the temperature of titration system. All data were fitted with the Origin 7.0 (MicroCal).

Microscale thermophoresis

MST analysis was performed using Nano Temper Monolith NT. LabelFree instrument (Nano Temper Technologies GmbH). Both the wild type XylE and XylE-WW protein were purified in MST buffer containing 25 mM MES pH 6.5, 150 mM NaCl and 0.056% Cymal-6. Gradient diluted sugars or inhibitors were mixed with XylE variants. The mixtures were loaded to MO-Z002 capillaries after 2 min incubation at room

temperature. MST measurements were performed using 20% MST power and 20% LED power. MST datasets were processed with the NT analysis 1.5.41 software (Nano Temper Technologies GmbH).

Supplementary Figure

Figure S1 | Structure-based design of XylE variant for intra-molecular crosslinking.

(a) Structural-guided identification of loci for cysteine mutations. Intracellular part cysteine pair (A152C and S396C) are presented as stick in outward open partially occluded XylE structure (PDB 4GBY). Extracellular part cysteine pair (V35C and E302C) are presented as stick in inward open partially occluded XylE structure (PDB 4JA3). **(b)** Location of DrICE protease cutting site in intracellular helix 3 (IC3). The intracellular helices 1-3 are colored orange. DrICE protease cutting site is colored marine. The detail of residue substitution illustrates on the right sequence. **(c)** Protein behaviors of EndoCC (A152C and S396C) and ExoCC (V35C and E302C) before crosslinking. **(d)** Transport activities of crosslinked XylE variants measured by proteoliposome-based transport assay. The transport activities of XylE variants are normalized against the wild type XylE. Control refers to protein-free liposome. Error bars represent s.d. The DrICE mutant is the wild type XylE only introduced with DrICE cutting site. A152C/S396C and V35C/E302C are EndoCC and ExoCC XylE before crosslinking. A152C/S396C crosslink and V35C/E302C crosslink represent the crosslinked EndoCC and ExoCC XylE.

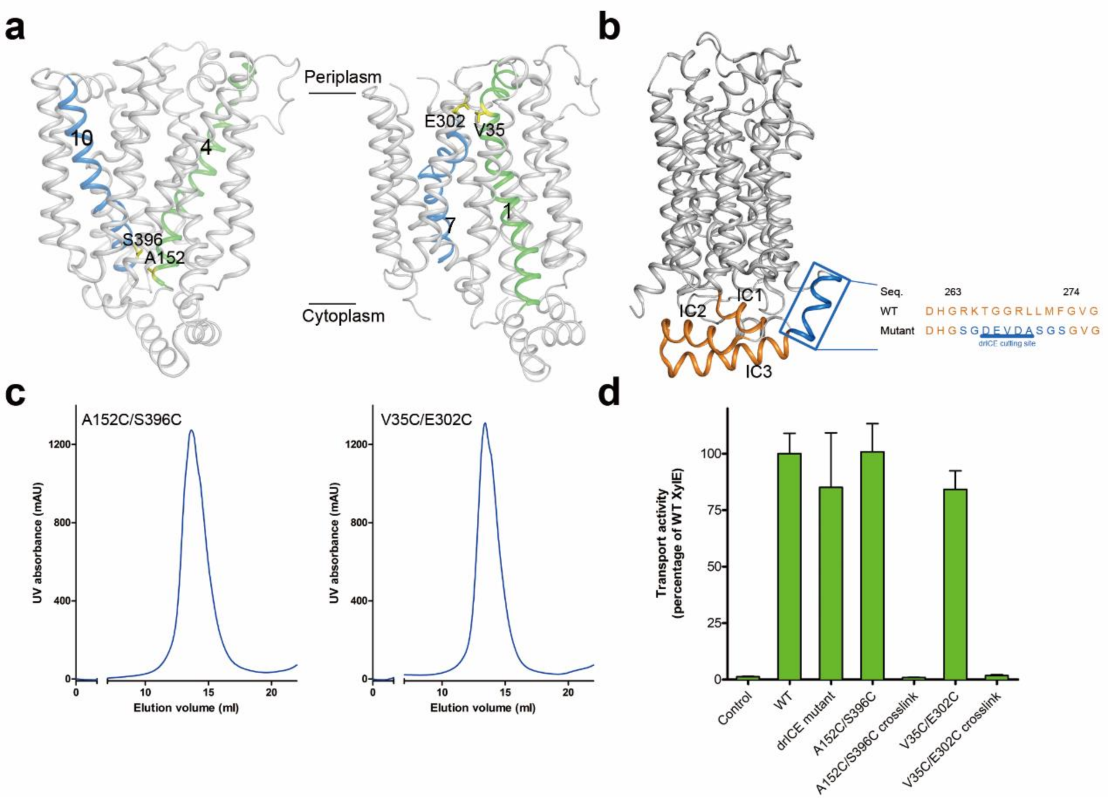
a
Periplasm
Cytoplasm
10
4
S396
A152
E302
V35
7
1
b
IC1
IC2
IC3
Seq. 263 274
WT D H G R K T G G R L L M F G V G
Mutant D H G S G D E V D A S G S G V G
c
A152C/S396C
V35C/E302C
UV absorbance (mAU)
Elution volume (ml)
d
Transport activity (percentage of WT XylE)
Control
WT
drICE mutant
A152C/S396C
A152C/S396C crosslink
V35C/E302C
V35C/E302C crosslink

Figure S2 | The raw data of ITC measurement of the binding affinity between xylose and XylE variants.

Binding affinity measurement between xylose and XylE variants through Isothermal Titration Calorimetry. The inner panels show the raw data of each injection. The area under each peak corresponds to the heat change of each injection. The outer panels show the function of total energy exchange against molar ratio of ligand to protein. The data was fitted by the MicroCal Origin 7.0 and presented as the solid lines in outer panels. All thermodynamic parameters are summarized in Supplementary Table S1.

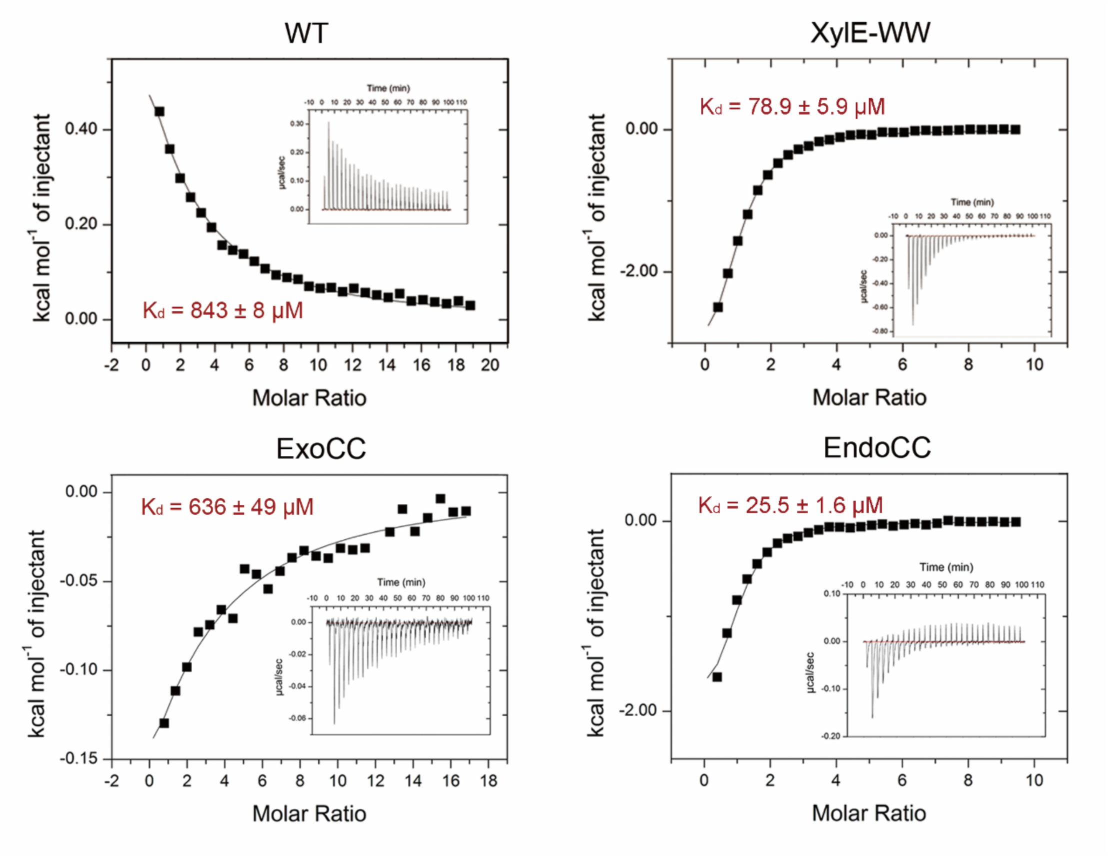

Figure S3 | Structural analyze of double tryptophan mutations in XylE-WW.

(a) Density map of double tryptophan mutations. G58W mutation locates between TM2 and TM11. L315W mutation locates between TM5 and TM8. Mutated residues are presented as stick and colored magenta. The density maps of TM2/TM11 and TM5/TM8, shown as blue mesh, are contoured at 1.0 σ. **(b)** Locations of double tryptophan mutations and substrate binding related residues. Xylose binding related residues are presented as stick and colored by elements. Double tryptophan mutations are colored magenta.

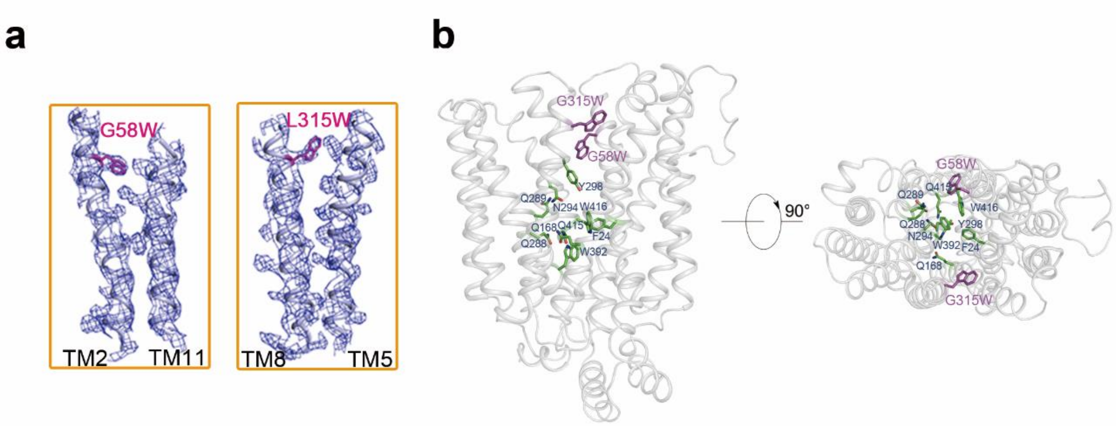

Figure S4 | The raw data of MST measurement of the binding affinity between GLUTs inhibitors and XylE variants.

Binding affinity measurement between GLUTs inhibitors and XylE variants through MicroScale Thermophoresis. The data was fitted by the NT analysis 1.5.41 and presented as the solid lines in outer panels.

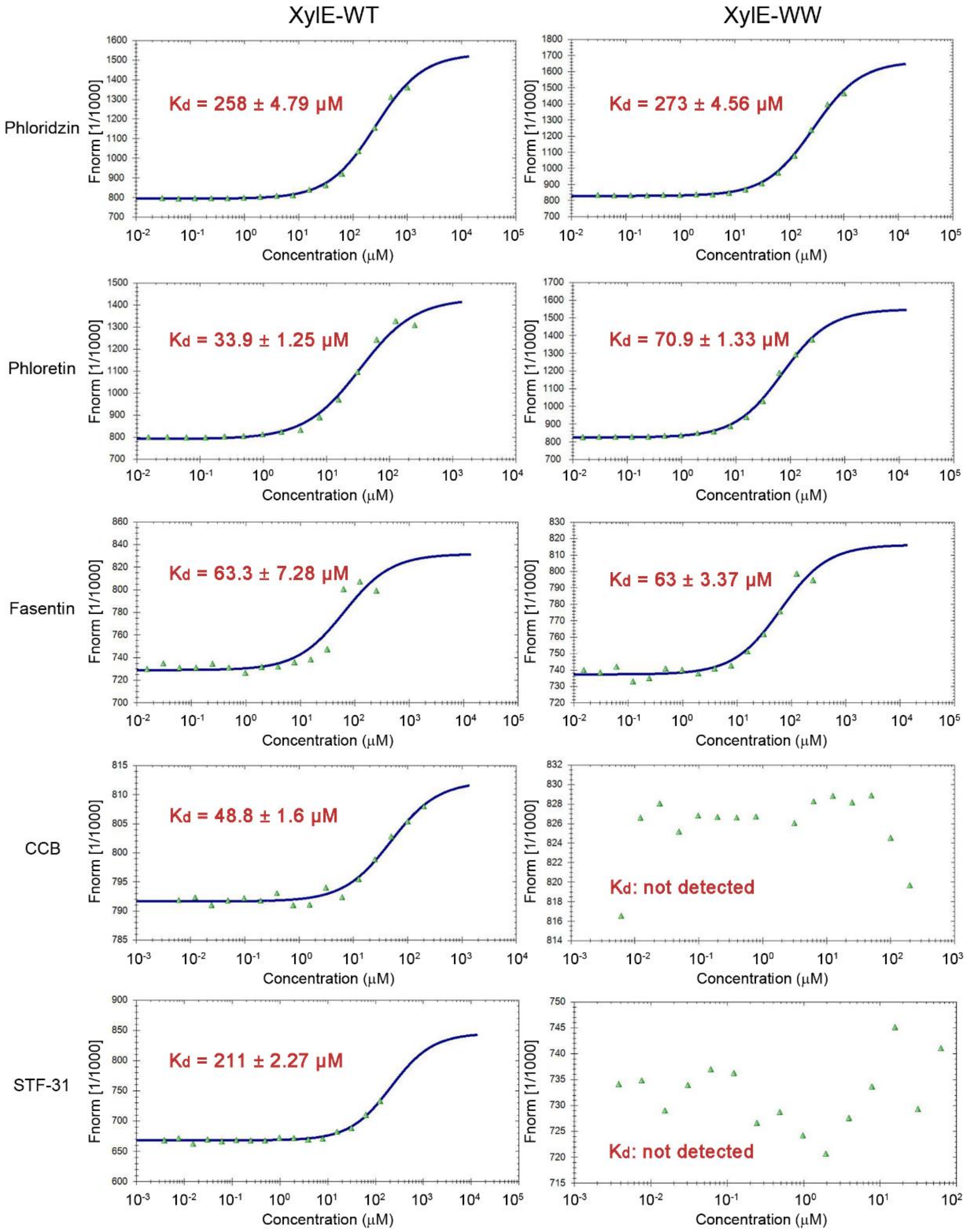

Tabel S1 | Thermodynamic parameters of ITC test

XylE	K_a (M^{-1})	ΔH ($kcal{\cdot}mol^{-1}$)	ΔG^1 ($kcal{\cdot}mol^{-1}$)	ΔS ($cal{\cdot}mol^{-1}{\cdot}k^{-1}$)	$T\Delta S^2$ ($kcal{\cdot}mol^{-1}$)
Wild type	$1.97x10^3$	2.95	-4.45	25.1	7.40
Crosslinked EndoCC	$3.96x10^4$	-1.38	-6.21	16.4	4.83
Crosslinked ExoCC	$1.59x10^3$	-0.81	-4.32	11.9	3.51
XylE-WW	$2.65x10^4$	-3.87	-5.97	7.12	2.10

1. $\Delta G = - RT \ln K_a$.

2. $T\Delta S = \Delta H - \Delta G$.

Table S2 | Statistics of data collection and refinement.

Protein	XylE-WW
Space Group	P 2_1 2_1 2_1
Unit Cell (Å)	89.08, 95.48, 106.84
Unit Cell (°)	90.00, 90.00, 90.00
Number of molecules in ASU	1
Wavelength (Å)	0.9793
Resolution (Å)	40~3.70 (3.83~3.70)
R_{merge} (%)	13.0 (90.5)
I/sigma	17.8 (2.4)
Completeness (%)	98.3 (99.5)
Number of measured reflections	54,967
Number of unique reflections	10,052
Redundancy	5.5 (5.6)
Wilson B factor (Å^2)	100.2
R-factor (%)	29.11
R_{free} (%)	32.96
Number of atoms	
Protein main chain	1900
Protein side chain	1741
Protein all atoms	3641
Water molecules	0
Other entities	0
All atoms	3641
Average B value (Å^2)	
Protein main chain	123.69
Protein side chain	124.59
Protein all atoms	124.12
Water molecules	0
Other entities	0
All atoms	124.12
R.m.s deviations from ideal values	
Bonds (Å)	0.006
Angle (°)	1.153
Ramachandran plot statistics (%)	
Most favorable	80.0
Additionally allowed	16.8
Generously allowed	3.2
Disallowed	0

Values in parentheses are for the highest resolution shell.

R_{merge}=$\Sigma_h\Sigma_i|I_{h,i}-I_h|/\Sigma_h\Sigma_i I_{h,i}$, where I_h is the mean intensity of the *i* observations of symmetry related reflections of *h*. R=$\Sigma|F_{obs}-F_{calc}|/\Sigma F_{obs}$, where F_{calc} is the calculated protein structure factor from the atomic model (R_{free} was calculated with 5% of the reflections selected).

Supplementary References

1 Sun, L. *et al.* Crystal structure of a bacterial homologue of glucose transporters GLUT1-4. *Nature* **490**, 361-366, doi:10.1038/nature11524 (2012).

2 Otwinowski, Z. & Minor, W. Processing of X-ray diffraction data collected in oscillation mode. *Method Enzymol* **276**, 307-326, doi:Doi 10.1016/S0076-6879(97)76066-X (1997).

3 Bailey, S. The Ccp4 Suite - Programs for Protein Crystallography. *Acta Crystallogr D* **50**, 760-763 (1994).

4 Mccoy, A. J. *et al.* Phaser crystallographic software. *J Appl Crystallogr* **40**, 658-674, doi:10.1107/S0021889807021206 (2007).

5 Emsley, P. & Cowtan, K. Coot: model-building tools for molecular graphics. *Acta Crystallogr D* **60**, 2126-2132, doi:10.1107/S0907444904019158 (2004).

6 Adams, P. D. *et al.* PHENIX: building new software for automated crystallographic structure determination. *Acta crystallographica. Section D, Biological crystallography* **58**, 1948-1954 (2002).
